# Revision Risk Following Total Hip Arthroplasty in Patients With Rheumatoid Arthritis: A Cohort Study From the Australian Orthopaedic Association National Joint Replacement Registry

**DOI:** 10.1111/ans.70797

**Published:** 2026-06-18

**Authors:** Owen Taylor‐Williams, Christopher J. Wall, Johannes Nossent, Carl Holder, Charles Inderjeeth

**Affiliations:** ^1^ Royal Perth Hospital Perth Western Australia Australia; ^2^ University of Western Australia Perth Western Australia Australia; ^3^ Darling Downs Health Toowoomba Queensland Australia; ^4^ University of Queensland Brisbane Queensland Australia; ^5^ Australian Orthopaedic Association National Joint Replacement Registry Adelaide South Australia Australia; ^6^ South Australian Health and Medical Research Institute Adelaide South Australia Australia; ^7^ Sir Charles Gairdner Osborne Park Hospital Group Perth Western Australia Australia

**Keywords:** dislocation, infection, loosening, rheumatoid arthritis, total hip arthroplasty

## Abstract

**Background:**

Total hip arthroplasty (THA) is one of the most successful orthopaedic procedures and provides good functional outcomes in both rheumatoid arthritis (RA) and osteoarthritis (OA) patients. However, RA patients face higher risks of adverse post‐operative outcomes. Despite recognising the unique risks of RA autoimmunity and drug therapy, there remains minimal RA‐specific evidence to guide arthroplasty surgeons.

**Methods:**

This study analysed the rate of all cause revision, and revision for infection, dislocation, periprosthetic fracture, and aseptic loosening for 3657 RA and 446 428 OA patients who underwent primary THA recorded in the Australian Orthopaedic National Joint Replacement Registry (AOANJRR).

**Results:**

Key findings of this study indicate that RA is associated with a younger age at THA, an increased risk of all‐cause revision (HR 1.35; 95% CI 1.16–1.56; *p* = 0.001), and an increased risk of revision for infection (HR 1.48; 95% CI 1.12–1.98; *p* = 0.006), dislocation (HR 1.87; 95% CI 1.45–2.42; *p* < 0.001), and early aseptic loosening (0–3 month; HR 2.81; 95% CI 1.50–5.26; *p* = 0.001). There was no difference in all‐cause revision for cemented, cementless, or hybrid THA fixation in RA patients.

**Conclusion:**

This study suggests that RA continues to be associated with worse post‐THA outcomes compared to OA. Further research is needed to develop strategies that improve perioperative management of inflammation and immune suppression to reduce the risk of post‐operative complications in RA patients undergoing THA.

## 1 Introduction

Rheumatoid arthritis (RA) is a common and prototypical autoimmune disease [1], that has the potential to cause devastating consequences to joint function. Since the introduction of methotrexate (MTX) as the prime conventional synthetic disease‐modifying antirheumatic drug (csDMARDs), there has been a rapid advancement of RA therapeutics towards biological disease‐modifying antirheumatic drugs (bDMARDs) and targeted synthetic disease‐modifying antirheumatic drugs (tsDMARDs) [2, 3, 4]. Increased understanding of RA pathophysiology has simultaneously led to new treatment paradigms advocating for early, intense therapy, which together with the expanded availability of therapeutic drugs has significantly reduced disease activity and long‐term consequences [5, 6, 7, 8, 9, 10, 11]. However, despite the improvements in controlling disease activity, the cumulative burden of long‐term disease exposure continues to result in patients requiring arthroplasty procedures to relieve pain and improve function [1, 12].

Total hip (THA) and total knee arthroplasty (TKA) are the two most common orthopaedic procedures in RA and have proven to be immensely successful [13, 14, 15], with RA patients achieving similar functional improvement as OA patients [1, 16, 17]. However, due to RA's effects on periarticular soft tissue and extra‐articular systems, RA patients face increased risks of complications and mortality post‐operatively [1, 16, 18, 19]. Improved understanding of RA has been associated with an evolving understanding of the need to adapt surgical strategies for these patients [1]. However, as highlighted by Zwartele et al. in a systematic review, the majority of the existing evidence for surgical technique and implant choice in RA THA is based on small, single‐centre studies, with dubious generalisability and lack of significant power [20]. Hence, surgeons' ability to adapt their technique and achieve optimal surgical outcomes in RA patients is limited [1]. The aim of this study was to determine whether RA patients have higher revision rates following primary THA compared to patients with OA, using national registry data [21].

## 2 Methods

The Australian Orthopaedic Association National Joint Replacement Registry (AOANJRR) commenced data collection in September 1999, achieving complete national implementation by mid‐2002 [22]. Since then, the AOANJRR has collected data on almost 100% of THA procedures performed in Australia. The diagnosis for which the THA is performed is recorded on a standardised form at the time of surgery (see data collection form in Figure S1). The diagnosis of RA or OA is assigned by the treating surgeon.

AOANJRR data is externally validated against patient‐level data provided by all Australian state and territory health departments. A sequential, multilevel matching process is used to identify any missing data, which is subsequently retrieved by contacting the relevant hospital. Each month, in conjunction with internal validation and data quality checks, all primary procedures are linked to any subsequent revision involving the same patient, same joint, and same side. Data are also matched annually with the Australian Government's National Death Index to obtain information on the date of death; linking revision and death to the primary procedure enables revision rates to be determined.

All patients who underwent primary conventional THA from 1 September 1999 to 31 December 2024 were identified (*n* = 720 817). Patients with a diagnosis other than RA or OA (*n* = 81 566), non‐highly cross‐linked polyethylene liners or metal‐on‐metal bearing surfaces (*n* = 185 112), modular neck implants (*n* = 3892), or constrained designs (*n* = 162) were excluded because the AOANJRR has previously identified an association with higher revision rates and these are no longer used in common practice for primary THA [21].

The primary outcome of interest in this study was all‐cause revision, with the secondary outcomes being revision for infection, dislocation, aseptic loosening, and periprosthetic fracture (PPF). Revision was defined as the removal, replacement, or addition of any device component [21].

Cumulative percent revision (CPR) was calculated by the Kaplan–Meier method, and compared between RA and OA patients for all‐cause revision, as well as revision for infection, dislocation, aseptic loosening, and PPF. Hazard ratios (HR) were calculated using Cox proportional hazards models, adjusted for age and sex. The assumption of proportional hazards was checked by testing for a significant interaction between each covariate and the log of time. Specifically, if the HR was not proportional over the entire time of observation, then a time varying model was used, which estimated a separate HR within each pre‐defined time period. Within each time period, the hazards are proportional. The AOANJRR uses a set algorithm which iteratively chooses time points until the assumption of proportional hazards was met for each time period. The time points are selected based on where the greatest change in hazard occurs between the two comparison groups, weighted by the number of events in that time period. If no specific time limit is specified, then HR are approximated for the lifetime of patients. Sub‐group analysis was performed for male and female patients and patients aged under 65 years and equal to or greater than 65 years. Revision rates and ratios are reported with 95% confidence intervals (CI).

Statistical analysis was performed using SAS software version 9.4 (SAS Institute, Cary, North Carolina, USA).

The AOANJRR is approved by the Commonwealth of Australia as a Federal Quality Assurance Activity under Part VC of the Health Insurance Act, 1973. All AOANJRR studies are conducted in accordance with ethical principles of research (Helsinki Declaration II). The AOANJRR is funded by the Commonwealth of Australia Department of Health. No other sources of funding were used in this study.

A total of 450 085 THA procedures were included, of which 3657 (0.8%) were in RA and 446 428 (99.2%) in OA patients. Overall, the mean age was 69.2 years, and 54.8% were female. The majority of patients were pre‐obese (36.3%) or obese (42.7%), and American Society of Anesthesiologists class (ASA) 2 (52.9%) or 3 (38.8%). Further demographic details are provided in Table 1.

**TABLE 1:** Demographics of patients who underwent primary total hip arthroplasty by primary diagnosis.

Variable		Osteoarthritis	Rheumatoid arthritis	TOTAL
Follow up years	Mean (SD)	6.6 (5)	7.6 (5.5)	6.6 (5)
	Median (IQR)	5.7 (2.5, 9.7)	6.5 (2.9, 11.2)	5.7 (2.5, 9.7)
	Minimum	0	0	0
	Maximum	24.7	24.7	24.7
Age	Mean (SD)	69.2 (10.3)	64.7 (13.7)	69.2 (10.3)
	Median (IQR)	70 (63, 77)	67 (58, 74)	70 (63, 77)
Age group	< 55	38 252 (8.6%)	662 (18.1%)	38 914 (8.6%)
	55–64	97 611 (21.9%)	881 (24.1%)	98 492 (21.9%)
	65–74	164 150 (36.8%)	1223 (33.4%)	165 373 (36.7%)
	≥ 75	146 415 (32.8%)	891 (24.4%)	147 306 (32.7%)
Sex	Male	202 353 (45.3%)	973 (26.6%)	203 326 (45.2%)
	Female	244 075 (54.7%)	2684 (73.4%)	246 759 (54.8%)
Procedure year	1999–2005	25 944	442	26 386 (5.86%)
	2006–2012	85 108	918	86 026 (19.11%)
	2013–2019	167 829	1250	169 079 (37.57%)
	2020–2024	167 547	1047	168 594 (37.46%)
Femoral fixation	Cementless	265 828 (59.5%)	1683 (46%)	267 511 (59.4%)
	Cemented	180 600 (40.5%)	1974 (54%)	182 574 (40.6%)
Femoral head size	< 32 mm	77 965 (17.5%)	992 (27.1%)	78 957 (17.5%)
	32 mm	165 638 (37.1%)	1488 (40.7%)	167 126 (37.1%)
	> 32 mm	202 818 (45.4%)	1177 (32.2%)	203 995 (45.3%)
ASA score^a^	1	22 686 (6.9%)	53 (2.4%)	22 739 (6.9%)
	2	173 912 (53%)	844 (37.7%)	174 756 (52.9%)
	3	126 926 (38.7%)	1272 (56.8%)	128 198 (38.8%)
	4	4759 (1.4%)	69 (3.1%)	4828 (1.5%)
	5	24 (0%)		24 (0%)
BMI^b^	Underweight (< 18.50)	2151 (0.8%)	38 (2%)	2189 (0.8%)
	Normal (18.50–24.99)	58 203 (20.3%)	484 (25.7%)	58 687 (20.3%)
	Pre obese (25.00–29.99)	104 098 (36.3%)	605 (32.2%)	104 703 (36.3%)
	Obese class 1 (30.00–34.99)	74 471 (26%)	456 (24.2%)	74 927 (26%)
	Obese class 2 (35.00–39.99)	32 282 (11.3%)	201 (10.7%)	32 483 (11.3%)
	Obese lass 3 (≥ 40.00)	15 544 (5.4%)	97 (5.2%)	15 641 (5.4%)
Total		446 428	3657	450 085

Abbreviations: ASA, American Society of Anesthesiologists; BMI, Body Mass Index (kg/m^2^); IQR, interquartile range; SD, standard deviation.

^a^ Excludes 119 358 procedures with unknown ASA Score.

^b^ Excludes 160 201 procedures with unknown BMI.

## 3 Results

RA THA patients, compared to OA patients, tended to be younger (median 67 years vs. 70 years), predominantly female (73.4% vs. 54.7%), proportionally underwent more cemented femoral fixation (54.0% vs. 40.5%), fell into ASA class 3 (56.8% vs. 38.7%), were more likely to be normal‐weight or underweight (27.7% vs. 21.1%), and were more likely to have a femoral head diameter of 32 mm or less (67.8% vs. 54.6%). The mean follow‐up was 6.6 years, with a slightly longer follow‐up in the RA group (7.6 years). Further demographic details are available in Table 1.

### 3.1 All Cause Revision

**FIGURE 1:**
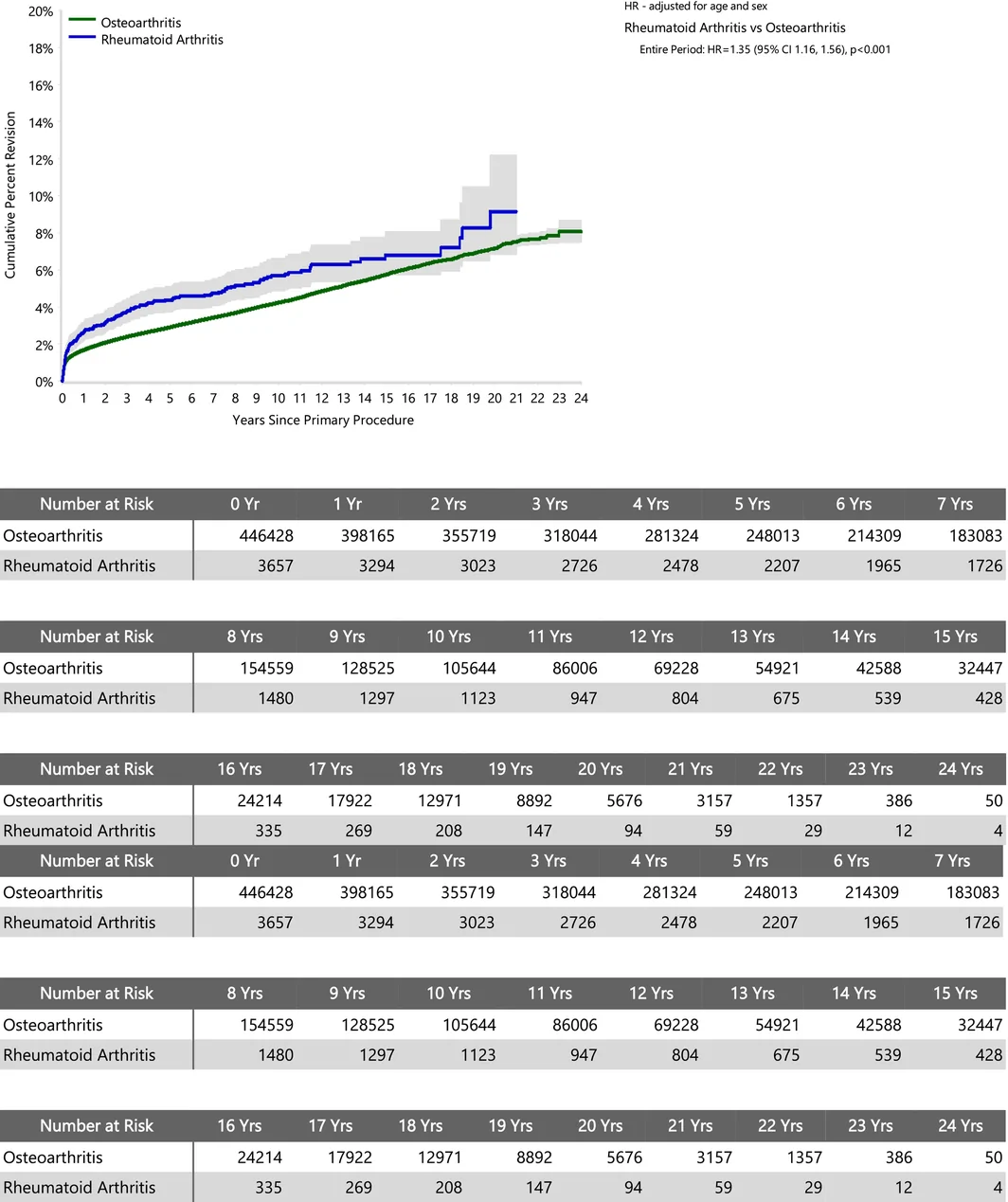
Cumulative percent all‐cause revision following primary total hip arthroplasty in patients with rheumatoid arthritis and osteoarthritis.

A total of 181 RA patients and 15 091 OA patients underwent a revision procedure during the study period. When adjusted for age and sex, RA was associated with a higher risk (hazard) of revision (HR 1.35; 95% CI 1.16–1.56; *p* = 0.001). See Figure 1.

### 3.2 Temporal Trends in All‐Cause Revision

Across all time periods, RA was associated with higher hazards of all‐cause revision, except for the time period from 2006 to 2012, where there was no difference between groups. Estimated HR were 1.41 (95% CI 1.00–1.99) in 1999–2005, 1.40 (95% CI 1.09–1.79) in 2013–2019, and 2.02 (1.49–2.73, *p* < 0.001) in 2020–2024. See Figures S2–S5.

### 3.3 Causes of Revision

Dislocation was the most common cause of revision in RA (33.7% of all revisions), while infection was the most common cause of revision in OA (25.5% of all revisions). When adjusted for age and sex, RA patients had a higher hazard of revision for infection (HR 1.48; 95% CI 1.12–1.98; *p* = 0.006) (Figure S6), dislocation/instability (HR 1.87; 95% CI 1.45–2.42 *p* < 0.001) (Figure S7), and early aseptic loosening (0–3 months; HR 2.81; 95% CI 1.50–5.26; *p* = 0.001), compared to OA patients (Figure S8). However, from 3 months onwards there was no difference in the hazards of revision for aseptic loosening between groups (HR 0.96; 95% CI 0.64–1.45; *p* = 0.852). There was no difference in the hazard ratios of revision for PPF between groups (Figure S9). See Table 2.

**TABLE 2:** Reasons for revision following primary total hip arthroplasty in patients with rheumatoid arthritis and osteoarthritis.

Revision diagnosis	Osteoarthritis			Rheumatoid arthritis		
	Number	% Primaries revised	% Revisions	Number	% Primaries revised	% Revisions
Infection	3845	0.9	25.5	48	1.3	26.5
Fracture	3500	0.8	23.2	28	0.8	15.5
Prosthesis dislocation/instability	3495	0.8	23.2	61	1.7	33.7
Loosening	2709	0.6	18.0	33	0.9	18.2
Pain	251	0.1	1.7	2	0.1	1.1
Leg length discrepancy	228	0.1	1.5	1	0.0	0.6
Malposition	210	0.0	1.4	2	0.1	1.1
Implant breakage stem	141	0.0	0.9	1	0.0	0.6
Lysis	121	0.0	0.8	1	0.0	0.6
Metal related pathology	84	0.0	0.6			
Incorrect sizing	80	0.0	0.5	1	0.0	0.6
Wear acetabular Insert	62	0.0	0.4			
Implant breakage acetabular insert	59	0.0	0.4			
Implant breakage acetabular	31	0.0	0.2	1	0.0	0.6
Heterotopic bone	25	0.0	0.2			
Tumour	22	0.0	0.1			
Implant breakage head	6	0.0	0.0			
Wear acetabulum	4	0.0	0.0			
Wear head	2	0.0	0.0			
Osteonecrosis				1	0.0	0.6
Synovitis	1	0.0	0.0			
Other	215	0.0	1.4	1	0.0	0.6
N Revision	15 091	3.4	100.0	181	4.9	100.0
N Primary	446 428			3657		

### 3.4 Risk Factors for Revision

#### 3.4.1 Age

Considering patients aged under 65 years at the time of THA, RA is associated with higher rates of all‐cause revision (HR 1.33, 95% CI 1.07–1.64, *p* = 0.008), revision for infection (HR 1.52, 95% CI 1.02–2.26, *p* = 0.037), and revision for dislocation (HR 1.58, 95% CI 1.05–2.37, *p* = 0.027). There was no difference in the rate of revision for aseptic loosening or PPF between RA and OA groups.

Patients aged greater than or equal to 65 years with RA had a higher rate of all‐cause revision (HR 1.37; 95% CI 1.11–1.68; *p* = 0.002), revision for infection (HR 1.52, 95% CI 1.01–2.30, *p* = 0.044), and revision for dislocation (HR 2.13, 95% CI 1.54–2.95, *p* < 0.001). There was no difference in the rate of revision for aseptic loosening or PPF between RA and OA groups.

#### 3.4.2 Sex

There was no difference in all‐cause revision rates between male RA and OA patients. Male RA patients had a higher rate of revision for dislocation (HR 1.97; 95% CI 1.14, 3.40; *p* = 0.015), compared to male OA patients. There was no difference in the rate of revision for infection, aseptic loosening, or PPF between male RA and OA patients.

Female RA patients, compared to female OA patients, had a higher rate of all‐cause revision (HR 1.36; 95% CI 1.15, 1.62; *p* = 0.001), revision for infection (HR 1.68; 95% CI 1.21, 2.33; *p* = 0.001), dislocation (HR 1.82; 95% CI 1.37, 2.43; *p* < 0.001), and early aseptic loosening (0–3 months; HR 3.26; 95% CI 1.73, 6.14; *p* < 0.001). From 3 months onwards, there was no difference in the rate of revision for aseptic loosening between female RA and OA patients. There was no difference in the rate of revision for PPF between female RA and OA patients.

### 3.5 Fixation

**FIGURE 2:**
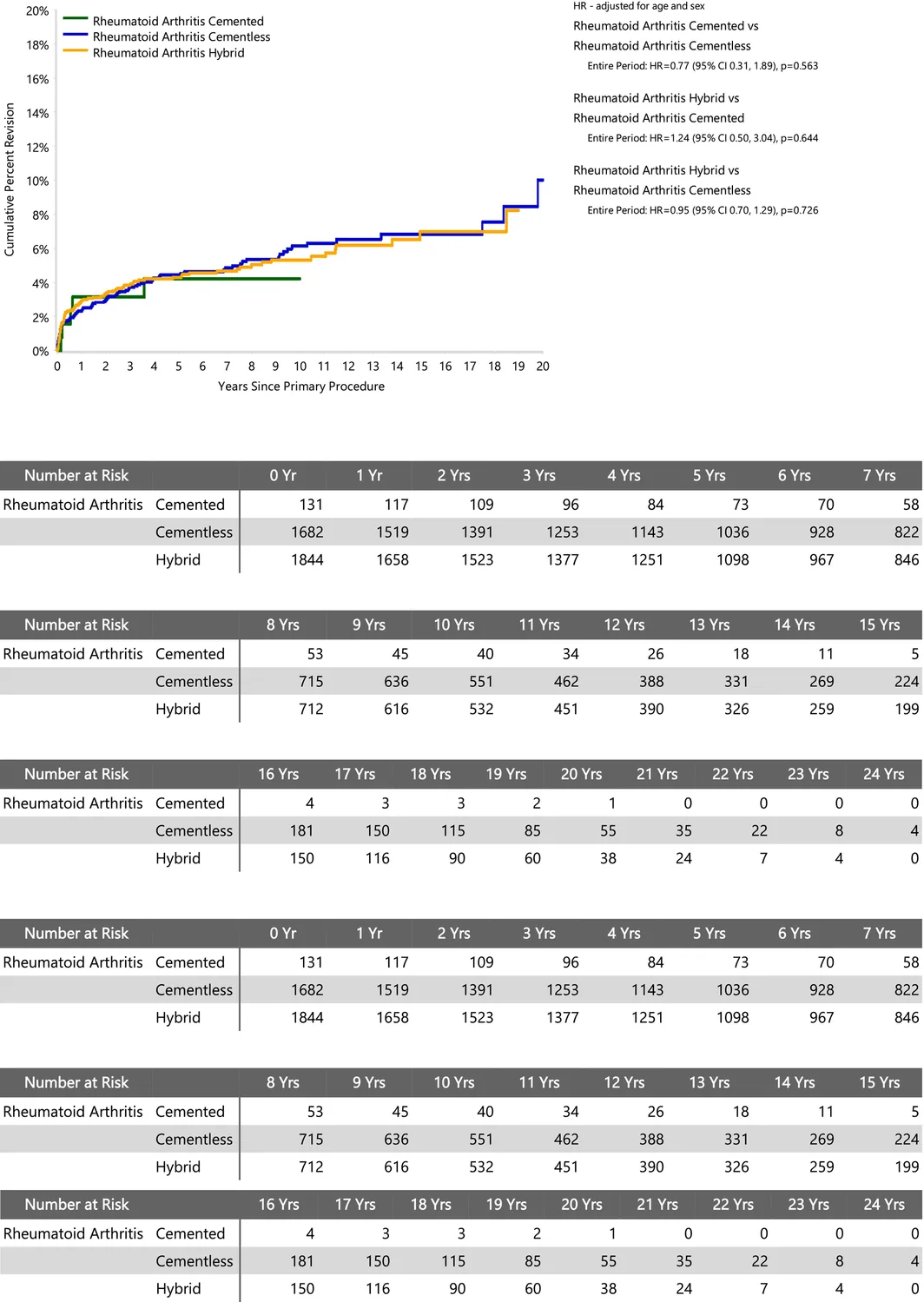
Cumulative percent all‐cause revision following primary total hip arthroplasty in patients with rheumatoid arthritis, by fixation type.

Utilisation of cemented (*n* = 115), cementless (*n* = 1514), or hybrid (*n* = 1640) fixation did not affect all‐cause revision following THA in RA patients. See Figure 2.

### 3.6 Femoral Head Size

There was no difference in the rate of all‐cause revision for RA and OA patients undergoing THA with femoral head size < 32 mm (*n* = 992) or > 32 mm (*n* = 1177) (*p* = 0.088 and *p* = 0.335, respectively). However, RA patients with a femoral head size of 32 mm (*n* = 1488) did have a higher rate of all‐cause revision (HR 1.53, 95% CI 1.23–1.91, *p* < 0.001) compared to OA patients.

## 4 Discussion

This national registry study comparing RA and OA THA outcomes in Australia fills a notable void in the literature identified in a previous systematic review [11]. Key findings are that RA patients who undergo primary THA have a higher rate of all‐cause revision and revision for infection, dislocation, and early aseptic loosening compared to patients with OA. Additionally, the choice of cemented, hybrid, or cementless fixation does not affect post‐THA survival in RA patients.

### 4.1 Overall Risk of Revision

Previous research papers have found that RA patients are at a higher risk of revision compared to OA [11]. However, it has been hypothesised that with newer DMARDs, advancement in perioperative care, and a better understanding of the RA disease process, the risk of revision may become comparable to the OA cohort [10, 11]. However, this study suggests that RA patients in the most recent decade continue to have a similar increased risk of revision to that of their counterparts 20+ years ago. The explanation for why the risk of revision for RA patients relative to OA has not improved is unclear, but it could be related to a trend toward operating on more unwell patients [21].

### 4.2 Infection

Consistent with previous data, RA appears to be associated with an increased risk of revision for infection. The epidemiological effect of RA on revision for infection appears similar to, or less than, that estimated in studies of RA patients on csDMARDs or in the era before bDMARDs [2, 3, 4, 16, 23, 24, 25, 26, 27, 28]. Hence, while this study is not able to directly estimate the effect of RA disease activity or pharmacological therapies, given that csDMARD and bDMARD therapy have been available throughout the time of data collection, this study appears to support the literature that increasing the use of csDMARDs and bDMARDs in the RA population, with current prophylactic strategies, does not result in a large increase in revision for infection [2, 3, 4, 16, 23, 24, 25, 26, 27, 28]. An alternative explanation is that this study underestimates the true number of post‐operative infections. For example, superficial surgical site infection was not considered in this analysis, because not all of these patients progress to requiring a revision operation. Moreover, a proportion of deep prosthetic joint infections may not be considered, because some surgeons do not revise minor components during debridement procedures. However, we would expect infection to be under‐reported to the same extent in the OA and RA cohorts.

### 4.3 Dislocation

RA is associated with a higher risk of revision for dislocation, with age over 65 at primary THA identified as a risk factor. Many studies have reported that RA patients are at a higher risk of dislocation following THA [29], with previous registry‐based studies estimating rates of revision between 1.3% [27] and 3.3% [30]. These rates underestimate the burden of dislocation in RA patients, with true rates estimated at over 15%, because most of these dislocations are managed without revision [31]. Nonetheless, the association of RA with increased revision for dislocation is a cause for concern because of the increased morbidity and mortality associated with revision procedures [32, 33, 34]. Potential reasons why RA patients are at an increased risk of dislocation include an inferior quality of soft tissues, weaker muscles, a higher risk of other physical impairments, protrusio acetabuli, and suboptimal hip abductor strength [27]. An alternative, less likely explanation might be a higher risk of implant mispositioning in RA patients [35], but we are unable to comment on this. The higher rates of dislocation evident in RA patients suggest that the use of dual mobility liners should be routinely considered in RA patients, especially if they are aged over 65 years.

### 4.4 Loosening

While previous studies have suggested that RA is associated with an increased risk of aseptic loosening [11], this analysis is the first to suggest that RA is associated with an increased risk of revision for early aseptic loosening, within 3 months of THA, but not after this time. This is significant because early aseptic loosening is associated with poor bone stock, insufficient initial fixation, and necrotic bone [36], all of which are correlated with RA disease activity [25]. Combined with the findings of previous studies that RA patients are at high risk of disease flares in the postoperative period [2, 16, 37], it might appear there is a need to consider emphasising the importance of intensive disease control in the early postoperative period, during a time when many DMARDs are withheld or underdosed, to achieve better THA outcomes. A recent study suggested that bisphosphonate use may reduce resorption of early necrotic bone and early aseptic loosening [36, 38]. These concepts were further supported in a recent systematic review, which suggested that bisphosphonate therapy be considered for up to 1 year post‐THA to promote early bony ingrowth and increase prosthesis longevity [38]. Overall, DMARDs' effects on bones, DMARDs' effects on prosthesis fixation, and antiosteoporotic therapies' effects on prosthesis remain limited, but early results suggest there is room for optimisation. Hence, there appears to be a need to continue investigating the optimal balance between immunosuppression, disease control, and anti‐osteoporotic therapy in the early post‐THA management of RA patients.

### 4.5 Fixation Technique

Whether cemented, cementless, or hybrid fixation has better outcomes in RA patients remains unresolved [39]. A recent systematic review concurs with classical teaching that a cemented technique is preferable [40]; however, our study demonstrated no difference in revision rates among the three fixation groups (cemented, hybrid, or uncemented). Hence, it would appear reasonable for surgeon preference to guide the use of fixation technique, until there is better evidence from randomised control trials.

### 4.6 Strengths and Limitations

This study has numerous strengths, including its large size and near complete ascertainment of THA cases over more than two decades. This has allowed us to detect differences between the RA and OA cohorts, which have previously been limited due to the low incidence of RA. Secondly, the prospective, observational nature of this study allows for a pragmatic understanding of how RA and OA impact prosthesis revision rates in the real world, outside of single‐centre cohorts or randomised control trials. Thirdly, we excluded non‐modern implant designs, improving the relevance of our results. However, we acknowledge several limitations to our study. The AOANJRR relies on operating theatre personnel to record the reason for THA, hence, the diagnosis of RA was based on recorded data at the time of operation by operating theatre staff and not necessarily verified by a surgeon or a rheumatologist or against the American College of Rheumatology standardised definition. As such, we suspect a proportion of RA cases may not have been recognised. Our selection criteria, only including cases with highly cross‐linked polyethylene liners, representing modern implant use in Australia, may be partially responsible for our apparently missing cases. Nonetheless, there are a few resulting effects that could bias our estimate of revision risk: first, the reassignment of RA cases to OA might increase the apparent incidence of complications in OA; however, given the large volume of OA cases in this study, the effect of this bias is likely minimal. Second, the reassignment of RA cases to OA would reduce the number of events in RA and, hence, the effect size and power to identify a significant result. Third, RA cases identified may be more severe, thereby biasing this study toward finding a greater association between RA and THA revision risk. As a result, the authors conclude that RA is associated with a higher risk of revision. However, this effect may be less apparent in studies focusing on RA patients with low disease severity or with other prosthesis designs. A further limitation was that the AOANJRR does not record specific data for RA therapeutics or disease activity of RA patients, nor data pertaining to other medical comorbidities. We acknowledge that other variables beyond patient age and sex affect revision rates following THA. Using data routinely collected by the AOANJRR, we conducted a sensitivity analysis including age, sex, bearing design (conventional versus dual mobility), year of surgery, femoral fixation (cemented versus cementless), head size, and hospital type (public versus private). The adjusted HR for all‐cause revision was 1.38 (95% CI 1.19–1.60; *p* < 0.001), which was very similar to the HR adjusted for age and sex alone (HR 1.35; 95% CI 1.16–1.56; *p* = 0.001). Similarly, we conducted a sensitivity analysis for the subset of patients with available ASA scores and BMI data. The HR for all‐cause revision, adjusted for age, sex, ASA score, and BMI, was 1.41 (95% CI 1.10–1.81; *p* = 0.007), which was also very similar to the HR adjusted for age and sex alone. The results of these sensitivity analyses add weight to the overall results of this study.

## 5 Conclusion

This national registry study has demonstrated that RA patients, compared with OA patients undergoing primary THA, have an increased risk of all‐cause revision, revision for infection, dislocation, and early aseptic loosening. From here, our data supports arthroplasty surgeons considering using dual mobility liners in RA to reduce dislocation risk, and researchers to review the outcomes of different implant fixation concepts and the role of bisphosphonate therapy post‐THA in RA patients. Evidence from randomised control trials would be ideal to make definitive conclusions; however, this may not be achievable in a real‐world setting, with variations in surgical expertise and preference, and patient‐related factors.

## Author Contributions

**Christopher J. Wall:** conceptualization, supervision, writing – review and editing, project administration. **Charles Inderjeeth:** writing – review and editing, supervision. **Johannes Nossent:** writing – review and editing, supervision. **Owen Taylor‐Williams:** conceptualization, investigation, writing – original draft, methodology, validation, writing – review and editing, project administration. **Carl Holder:** visualization, software, formal analysis, data curation.

## Funding

The authors have nothing to report.

## Conflicts of Interest

The authors declare no conflicts of interest.

## Supporting information


**Figure S1:** AOANJRR data collection form for total hip arthroplasty procedures.
**Figure S2:** Cumulative percent revision of primary total conventional hip arthroplasty by primary diagnosis for procedures performed between 1999 and 2005 (All‐cause revision).
**Figure S3:** Cumulative percent revision of primary total conventional hip arthroplasty by primary diagnosis for procedures performed between 2006 and 2012 (All‐cause revision).
**Figure S4:** Cumulative percent revision of primary total conventional hip arthroplasty by primary diagnosis for procedures performed between 2013 and 2019 (All‐cause revision).
**Figure S5:** Cumulative percent revision of primary total conventional hip arthroplasty by primary diagnosis for procedures performed between 2020 and 2024 (All‐cause revision).
**Figure S6:** Cumulative percent revision of primary total conventional hip arthroplasty by primary diagnosis (Revision for dislocation).
**Figure S7:** Cumulative percent revision of primary total conventional hip arthroplasty by primary diagnosis (Revision for infection).
**Figure S8:** Cumulative percent revision of primary total conventional hip arthroplasty by primary diagnosis (Revision for aseptic loosening).

## Data Availability

The data that supports the findings of this study are available in the Supporting Information of this article.
