# Supplementary material for: Revision Risk Following Total Hip Arthroplasty in Patients With Rheumatoid Arthritis: A Cohort Study From the Australian Orthopaedic Association National Joint Replacement Registry
Source: ANZ J Surg. 2026 Jun 18;96(9):2388–97. doi: 10.1111/ans.70797 (PMC13602448; doi:10.1111/ans.70797)
Supplement: Supplementary file 1 [file ANS-96-2388-s001.docx]

**S1: AOANJRR data collection form for total hip arthroplasty procedures**

**S2: Cumulative percent revision of primary total conventional hip arthroplasty by primary diagnosis for procedures performed between 1999 and 2005 (All-cause revision)**

| **Number at Risk** |  | **0 Yr** | **1 Yr** | **2 Yrs** | **3 Yrs** | **4 Yrs** | **5 Yrs** | **6 Yrs** | **7 Yrs** |
| --- | --- | --- | --- | --- | --- | --- | --- | --- | --- |
| Osteoarthritis | 1999-2005 | 25944 | 25180 | 24608 | 23991 | 23304 | 22564 | 21691 | 20742 |
| Rheumatoid Arthritis | 1999-2005 | 442 | 421 | 409 | 390 | 370 | 352 | 334 | 319 |

| **Number at Risk** |  | **8 Yrs** | **9 Yrs** | **10 Yrs** | **11 Yrs** | **12 Yrs** | **13 Yrs** | **14 Yrs** | **15 Yrs** |
| --- | --- | --- | --- | --- | --- | --- | --- | --- | --- |
| Osteoarthritis | 1999-2005 | 19838 | 18798 | 17775 | 16760 | 15694 | 14607 | 13563 | 12514 |
| Rheumatoid Arthritis | 1999-2005 | 298 | 288 | 277 | 259 | 250 | 234 | 225 | 207 |

| **Number at Risk** |  | **16 Yrs** | **17 Yrs** | **18 Yrs** | **19 Yrs** | **20 Yrs** | **21 Yrs** | **22 Yrs** | **23 Yrs** | **24 Yrs** |
| --- | --- | --- | --- | --- | --- | --- | --- | --- | --- | --- |
| Osteoarthritis | 1999-2005 | 11536 | 10645 | 9752 | 8892 | 5676 | 3157 | 1357 | 386 | 50 |
| Rheumatoid Arthritis | 1999-2005 | 190 | 178 | 165 | 147 | 94 | 59 | 29 | 12 | 4 |

**S3: Cumulative percent revision of primary total conventional hip arthroplasty by primary diagnosis for procedures performed between 2006 and 2012 (All-cause revision)**

| **Number at Risk** |  | **0 Yr** | **1 Yr** | **2 Yrs** | **3 Yrs** | **4 Yrs** | **5 Yrs** | **6 Yrs** | **7 Yrs** | **8 Yrs** |
| --- | --- | --- | --- | --- | --- | --- | --- | --- | --- | --- |
| Osteoarthritis | 2006-2012 | 85108 | 82909 | 81278 | 79417 | 77320 | 75024 | 72329 | 69424 | 66392 |
| Rheumatoid Arthritis | 2006-2012 | 918 | 880 | 866 | 841 | 814 | 789 | 766 | 729 | 674 |

| **Number at Risk** |  | **9 Yrs** | **10 Yrs** | **11 Yrs** | **12 Yrs** | **13 Yrs** | **14 Yrs** | **15 Yrs** | **16 Yrs** | **17 Yrs** | **18 Yrs** |
| --- | --- | --- | --- | --- | --- | --- | --- | --- | --- | --- | --- |
| Osteoarthritis | 2006-2012 | 63256 | 60052 | 56818 | 53534 | 40314 | 29025 | 19933 | 12678 | 7277 | 3219 |
| Rheumatoid Arthritis | 2006-2012 | 653 | 623 | 588 | 554 | 441 | 314 | 221 | 145 | 91 | 43 |

**S4: Cumulative percent revision of primary total conventional hip arthroplasty by primary diagnosis for procedures performed between 2013 and 2019 (All-cause revision)**

| **Number at Risk** |  | **0 Yr** | **1 Yr** | **2 Yrs** | **3 Yrs** | **4 Yrs** | **5 Yrs** | **6 Yrs** | **7 Yrs** | **8 Yrs** | **9 Yrs** | **10 Yrs** | **11 Yrs** |
| --- | --- | --- | --- | --- | --- | --- | --- | --- | --- | --- | --- | --- | --- |
| Osteoarthritis | 2013-2019 | 167829 | 163382 | 160796 | 157749 | 154297 | 150425 | 120289 | 92917 | 68329 | 46471 | 27817 | 12428 |
| Rheumatoid Arthritis | 2013-2019 | 1250 | 1201 | 1178 | 1146 | 1106 | 1066 | 865 | 678 | 508 | 356 | 223 | 100 |

**S5: Cumulative percent revision of primary total conventional hip arthroplasty by primary diagnosis for procedures performed between 2020 and 2024 (All-cause revision)**

| **Number at Risk** |  | **0 Yr** | **1 Yr** | **2 Yrs** | **3 Yrs** | **4 Yrs** |
| --- | --- | --- | --- | --- | --- | --- |
| Osteoarthritis | 2020-2024 | 167547 | 126694 | 89037 | 56887 | 26403 |
| Rheumatoid Arthritis | 2020-2024 | 1047 | 792 | 570 | 349 | 188 |

**S6: Cumulative percent revision of primary total conventional hip arthroplasty by primary diagnosis (Revision for dislocation)**

| **Number at Risk** | **0 Yr** | **1 Yr** | **2 Yrs** | **3 Yrs** | **4 Yrs** | **5 Yrs** | **6 Yrs** | **7 Yrs** |
| --- | --- | --- | --- | --- | --- | --- | --- | --- |
| Osteoarthritis | 446428 | 398165 | 355719 | 318044 | 281324 | 248013 | 214309 | 183083 |
| Rheumatoid Arthritis | 3657 | 3294 | 3023 | 2726 | 2478 | 2207 | 1965 | 1726 |

| **Number at Risk** | **8 Yrs** | **9 Yrs** | **10 Yrs** | **11 Yrs** | **12 Yrs** | **13 Yrs** | **14 Yrs** | **15 Yrs** |
| --- | --- | --- | --- | --- | --- | --- | --- | --- |
| Osteoarthritis | 154559 | 128525 | 105644 | 86006 | 69228 | 54921 | 42588 | 32447 |
| Rheumatoid Arthritis | 1480 | 1297 | 1123 | 947 | 804 | 675 | 539 | 428 |

| **Number at Risk** | **16 Yrs** | **17 Yrs** | **18 Yrs** | **19 Yrs** | **20 Yrs** | **21 Yrs** | **22 Yrs** | **23 Yrs** | **24 Yrs** |
| --- | --- | --- | --- | --- | --- | --- | --- | --- | --- |
| Osteoarthritis | 24214 | 17922 | 12971 | 8892 | 5676 | 3157 | 1357 | 386 | 50 |
| Rheumatoid Arthritis | 335 | 269 | 208 | 147 | 94 | 59 | 29 | 12 | 4 |

**S7: Cumulative percent revision of primary total conventional hip arthroplasty by primary diagnosis (Revision for infection)**

| **Number at Risk** | **0 Yr** | **1 Yr** | **2 Yrs** | **3 Yrs** | **4 Yrs** | **5 Yrs** | **6 Yrs** | **7 Yrs** | **8 Yrs** | **9 Yrs** | **10 Yrs** |
| --- | --- | --- | --- | --- | --- | --- | --- | --- | --- | --- | --- |
| Osteoarthritis | 230738 | 193797 | 163614 | 134128 | 107657 | 80794 | 56010 | 33886 | 14786 | 49 | 10 |
| Rheumatoid Arthritis | 1509 | 1279 | 1068 | 909 | 715 | 535 | 370 | 220 | 99 | 0 | 0 |

| **Number at Risk** | **11 Yrs** | **12 Yrs** | **13 Yrs** | **14 Yrs** | **15 Yrs** | **16 Yrs** | **17 Yrs** | **18 Yrs** | **19 Yrs** | **20 Yrs** | **21 Yrs** |
| --- | --- | --- | --- | --- | --- | --- | --- | --- | --- | --- | --- |
| Osteoarthritis | 5 | 2 | 1 | 1 | 1 | 1 | 1 | 1 | 1 | 1 | 1 |
| Rheumatoid Arthritis | 0 | 0 | 0 | 0 | 0 | 0 | 0 | 0 | 0 | 0 | 0 |

**S8: Cumulative percent revision of primary total conventional hip arthroplasty by primary diagnosis (Revision for aseptic loosening)**

| **Number at Risk** | **0 Yr** | **1 Yr** | **2 Yrs** | **3 Yrs** | **4 Yrs** | **5 Yrs** | **6 Yrs** | **7 Yrs** |
| --- | --- | --- | --- | --- | --- | --- | --- | --- |
| Osteoarthritis | 446428 | 398165 | 355719 | 318044 | 281324 | 248013 | 214309 | 183083 |
| Rheumatoid Arthritis | 3657 | 3294 | 3023 | 2726 | 2478 | 2207 | 1965 | 1726 |

| **Number at Risk** | **8 Yrs** | **9 Yrs** | **10 Yrs** | **11 Yrs** | **12 Yrs** | **13 Yrs** | **14 Yrs** | **15 Yrs** |
| --- | --- | --- | --- | --- | --- | --- | --- | --- |
| Osteoarthritis | 154559 | 128525 | 105644 | 86006 | 69228 | 54921 | 42588 | 32447 |
| Rheumatoid Arthritis | 1480 | 1297 | 1123 | 947 | 804 | 675 | 539 | 428 |

| **Number at Risk** | **16 Yrs** | **17 Yrs** | **18 Yrs** | **19 Yrs** | **20 Yrs** | **21 Yrs** | **22 Yrs** | **23 Yrs** | **24 Yrs** |
| --- | --- | --- | --- | --- | --- | --- | --- | --- | --- |
| Osteoarthritis | 24214 | 17922 | 12971 | 8892 | 5676 | 3157 | 1357 | 386 | 50 |
| Rheumatoid Arthritis | 335 | 269 | 208 | 147 | 94 | 59 | 29 | 12 | 4 |

**S9: Cumulative percent revision of primary total conventional hip arthroplasty by primary diagnosis (Revision for fracture)**

| **Number at Risk** | **0 Yr** | **1 Yr** | **2 Yrs** | **3 Yrs** | **4 Yrs** | **5 Yrs** | **6 Yrs** | **7 Yrs** |
| --- | --- | --- | --- | --- | --- | --- | --- | --- |
| Osteoarthritis | 446428 | 398165 | 355719 | 318044 | 281324 | 248013 | 214309 | 183083 |
| Rheumatoid Arthritis | 3657 | 3294 | 3023 | 2726 | 2478 | 2207 | 1965 | 1726 |

| **Number at Risk** | **8 Yrs** | **9 Yrs** | **10 Yrs** | **11 Yrs** | **12 Yrs** | **13 Yrs** | **14 Yrs** | **15 Yrs** |
| --- | --- | --- | --- | --- | --- | --- | --- | --- |
| Osteoarthritis | 154559 | 128525 | 105644 | 86006 | 69228 | 54921 | 42588 | 32447 |
| Rheumatoid Arthritis | 1480 | 1297 | 1123 | 947 | 804 | 675 | 539 | 428 |

| **Number at Risk** | **16 Yrs** | **17 Yrs** | **18 Yrs** | **19 Yrs** | **20 Yrs** | **21 Yrs** | **22 Yrs** | **23 Yrs** | **24 Yrs** |
| --- | --- | --- | --- | --- | --- | --- | --- | --- | --- |
| Osteoarthritis | 24214 | 17922 | 12971 | 8892 | 5676 | 3157 | 1357 | 386 | 50 |
| Rheumatoid Arthritis | 335 | 269 | 208 | 147 | 94 | 59 | 29 | 12 | 4 |
